# Supplementary material for: Perturbation-based trunk stabilization training in elite rowers: A pilot study
Source: PLoS One. 2022 May 19;17(5):e0268699. doi: 10.1371/journal.pone.0268699 (PMC9119454; doi:10.1371/journal.pone.0268699)
Supplement: S2 File — Study: Perturbation-based Trunk Stabilization Exercise in Elite Rowers. (PDF) [file pone.0268699.s002.pdf]

CERT – Consensus on Exercise Reporting Template

Study: Perturbation-based Trunk Stabilization Exercise in Elite Rowers

Section / Topic	Item #	Checklist item	Location Primary paper	† Other
**WHAT: materials**	1	Detailed description of the type of exercise equipment (e.g. weights, exercise equipment such as machines, treadmill, bicycle ergometer etc)	Methods – Intervention	OSF
**WHO: provider**	2	Detailed description of the qualifications, teaching/supervising expertise, and/or training undertaken by the exercise instructor	Methods – Intervention	OSF
**HOW: delivery**	3	Describe whether exercises are performed individually or in a group	Methods – Intervention	OSF
	4	Describe whether exercises are supervised or unsupervised and how they are delivered	Methods – Intervention	OSF
	5	Detailed description of how adherence to exercise is measured and reported	-	OSF
	6	Detailed description of motivation strategies	-	OSF
	7a	Detailed description of the decision rule(s) for determining exercise progression	Methods – Intervention	OSF
	7b	Detailed description of how the exercise program was progressed	Methods – Intervention	OSF
	8	Detailed description of each exercise to enable replication (e.g. photographs, illustrations , video etc)	%	OSF
	9	Detailed description of any home program component (e.g. other exercises, stretching etc)	-	OSF
	10	Describe whether there are any non-exercise components (e.g. education, cognitive behavioural therapy, massage etc)	-	OSF
	11	Describe the type and number of adverse events that occurred during exercise	Results	OSF
**WHERE: location**	12	Describe the setting in which the exercises are performed	Methods – Study Design	OSF
**WHEN, HOW MUCH: dosage**	13	Detailed description of the exercise intervention including, but not limited to, number of exercise repetitions/sets/sessions, session duration, intervention/program duration etc	Methods – Intervention	OSF
**TAILORING: what, how**	14a	Describe whether the exercises are generic (one size fits all) or tailored whether tailored to the individual	Methods – Intervention	OSF
	14b	Detailed description of how exercises are tailored to the individual	-	OSF
	15	Describe the decision rule for determining the starting level at which people commence an exercise program (such as beginner, intermediate, advanced etc)	-	OSF
**HOW WELL: planned, actual**	16a	Describe how adherence or fidelity to the exercise intervention is assessed/measured	-	OSF
	16b	Describe the extent to which the intervention was delivered as planned	Results	OSF
