# Supplementary material for: Perturbation-based trunk stabilization training in elite rowers: A pilot study
Source: PLoS One. 2022 May 19;17(5):e0268699. doi: 10.1371/journal.pone.0268699 (PMC9119454; doi:10.1371/journal.pone.0268699)
Supplement: S3 File — Modifications of the extension appear in italics and blue. (PDF) [file pone.0268699.s003.pdf]

2017 CONSORT checklist of information to include when reporting a randomized trial assessing nonpharmacologic treatments (NPTs)*. Modifications of the extension appear in italics and blue.

Section/Topic Item	Check list item no.	CONSORT item	Extension for NPT trials	Rep on page No
**Title and abstract**				
	1a	Identification as a randomized trial in the title		n/a
	1b	Structured summary of trial design, methods, results, and conclusions (for specific guidance see CONSORT for abstracts)	*Refer to CONSORT extension for abstracts for NPT trials*	*1*
**Introduction**				
Background and objectives	2a	Scientific background and explanation of rationale		3
	2b	Specific objectives or hypotheses		
**Methods**				
Trial design	3a	Description of trial design (such as parallel, factorial) including allocation ratio	When applicable, how care providers were allocated to each trial group	5
	3b	Important changes to methods after trial commencement (such as eligibility criteria), with reasons		6
Participants	4a	Eligibility criteria for participants	When applicable, eligibility criteria for centers and for *care providers*	6
	4b	Settings and locations where the data were collected		5
Interventions†	5	The interventions for each group with sufficient details to allow replication, including how and when they were actually administered	Precise details of both the experimental treatment and comparator	6-7 OSF
	5a		Description of the different components of the interventions and, when applicable, description of the procedure for tailoring the interventions to individual participants.	6-7 OSF
	5b		Details *of whether and* how the interventions were standardized.	6-7 OSF
	5c.		Details *of whether and* how adherence of care providers to the protocol was assessed or enhanced	6-7 OSF
	5d		*Details of whether and how adherence of participants to interventions was assessed or enhanced*	6-7 OSF
Outcomes	6a	Completely defined pre-specified primary and secondary outcome measures, including how and when they were assessed		**7-9**
	6b	Any changes to trial outcomes after the trial commenced, with reasons		**n/a**
Sample size	7a	How sample size was determined	When applicable, details of whether and how the clustering by care providers or centers was addressed	5 and 18

Cite as: *Boutron I, Altman DG, Moher D, Schulz KF, Ravaud P. CONSORT Statement for Randomized Trials of Nonpharmacologic Treatments: A 2017 Update and a CONSORT Extension for Nonpharmacologic Trial Abstracts. Annals of Internal Medicine. 2017 Jul 4;167(1):40–7.*

Section/Topic Item	Check list item no.	CONSORT item	Extension for NPT trials	Rep on page No
	7b	When applicable, explanation of any interim analyses and stopping guidelines		n/a
Randomization:				
- Sequence generation	8a	Method used to generate the random allocation sequence		n/a
	8b	Type of randomization; details of any restriction (such as blocking and block size)		n/a
- Allocation concealment mechanism	9	Mechanism used to implement the random allocation sequence (such as sequentially numbered containers), describing any steps taken to conceal the sequence until interventions were assigned		n/a
- Implementation	10	Who generated the random allocation sequence, who enrolled participants, and who assigned participants to interventions		n/a
Blinding	11a	If done, who was blinded after assignment to interventions (for example, participants, care providers, those assessing outcomes) and how	~~Whether or not those administering co-interventions were blinded to group assignment~~<br>If done, who was blinded after assignment to interventions (e.g., participants, care providers, *those administering co-interventions,* those assessing outcomes) and how	n/a
	11b	If relevant, description of the similarity of interventions	~~If blinded, method of blinding and description of the similarity of interventions~~	
	11c		*If blinding was not possible, description of any attempts to limit bias*	n/a
Statistical methods	12a	Statistical methods used to compare groups for primary and secondary outcomes	When applicable, details of whether and how the clustering by care providers or centers was addressed	10
	12b	Methods for additional analyses, such as subgroup analyses and adjusted analyses		10
**Results**				
Participant flow (a diagram is strongly recommended)	13a	For each group, the numbers of participants who were randomly assigned, received intended treatment, and were analyzed for the primary outcome	The number of care providers or centers performing the intervention in each group and the number of patients treated by each care provider or in each center	6
	13b	For each group, losses and exclusions after randomization, together with reasons		6
	13c		*For each group, the delay between randomization and the initiation of the intervention*	*n/a*

Cite as: *Boutron I, Altman DG, Moher D, Schulz KF, Ravaud P. CONSORT Statement for Randomized Trials of Nonpharmacologic Treatments: A 2017 Update and a CONSORT Extension for Nonpharmacologic Trial Abstracts. Annals of Internal Medicine. 2017 Jul 4;167(1):40–7.*

Section/Topic Item	Check list item no.	CONSORT item	Extension for NPT trials	Rep on page No
	new		Details of the experimental treatment and comparator as they were implemented	
Recruitment	14a	Dates defining the periods of recruitment and follow-up		**5**
	14b	Why the trial ended or was stopped		
Baseline data	15	A table showing baseline demographic and clinical characteristics for each group	When applicable, a description of care providers (case volume, qualification, expertise, etc.) and centers (volume) in each group.	11
Numbers analyzed	16	For each group, number of participants (denominator) included in each analysis and whether the analysis was by original assigned groups		11
Outcomes and estimation	17a	For each primary and secondary outcome, results for each group, and the estimated effect size and its precision (such as 95% confidence interval)		11-13
	17b	For binary outcomes, presentation of both absolute and relative effect sizes is recommended		n/a
Ancillary analyses	18	Results of any other analyses performed, including subgroup analyses and adjusted analyses, distinguishing pre-specified from exploratory		11-14
Harms	19	All important harms or unintended effects in each group (for specific guidance see CONSORT for harms)		11
**Discussion**				
Limitations	20	Trial limitations, addressing sources of potential bias, imprecision, and, if relevant, multiplicity of analyses	In addition, take into account the choice of the comparator, lack of or partial blinding, and unequal expertise of care providers or centers in each group	18
Generalizability	21	Generalizability (external validity, applicability) of the trial findings	Generalizability (external validity) of the trial findings according to the intervention, comparators, patients, and care providers and centers involved in the trial	18
Interpretation	22	Interpretation consistent with results, balancing benefits and harms, and considering other relevant evidence		15-18
**Other information**				
Registration	23	Registration number and name of trial registry		5
Protocol	24	Where the full trial protocol can be accessed, if available		
Funding	25	Sources of funding and other support (such as supply of drugs), role of funders		19

**Additions or modifications to the 2010 CONSORT checklist. CONSORT = Consolidated Standards of Reporting Trials*
†The items 5, 5a, 5b, 5c, 5d are consistent with the Template for Intervention Description and Replication (TIDieR) checklist

Cite as: *Boutron I, Altman DG, Moher D, Schulz KF, Ravaud P. CONSORT Statement for Randomized Trials of Nonpharmacologic Treatments: A 2017 Update and a CONSORT Extension for Nonpharmacologic Trial Abstracts. Annals of Internal Medicine. 2017 Jul 4;167(1):40–7.*
