# Supplementary material for: Perturbation-based trunk stabilization training in elite rowers: A pilot study
Source: PLoS One. 2022 May 19;17(5):e0268699. doi: 10.1371/journal.pone.0268699 (PMC9119454; doi:10.1371/journal.pone.0268699)
Supplement: S4 File — (PDF) [file pone.0268699.s004.pdf]

RUHR-UNIVERSITÄT BOCHUM | Faculty of Sports Science|
Bochum44780 | Germany

Faculty of Sport Science Ethics Committee
The chairman
Building: SW Health Campus
North 10
44801 Bochum

[redacted]

Mr. Robin Schäfer

Teaching and research area: Sports Medicine and Sports Nutrition
Faculty of Sports Science

Ruhr University Bochum

Bochum44780

Date
07.05.2018

Dear Mr. Schäfer,

The Ethics Committee of the Faculty of Sport Science has approved your application for the project with the title
"Feasibility and acceptance of a neuromuscular training concept in elite rowing athletes."
examined in the meantime.

The Commission considers your project to be ethically unobjectionable.

With kind regards

[redacted]
Chairman of the Ethics Committee of the Faculty of Sports Science

Request for opinion of the Ethics Committee of the Faculty of Sports Science (EKS) of the Ruhr-University Bochum

1. Title of the research project

"Feasibility and acceptance of a neuromuscular training concept in elite rowing athletes."

2. Name and contact details of the person making the application:

Last name, first name: Schäfer, RobinDienstbez: wissenschaftlicher

MitarbeiterTeaching and research area: Sports Medicine and Sports Nutrition

E-mail address: robin.schaefer@rub.de

Phone number: 0234 /32 - 22080

3. Brief summary of the research project

Objectives, collectives, methodological approach; max. words250

Within the framework of the long-term research project "Ran Rücken", the developed concepts of neuromuscular training are to be transfe- rred into the general population as well as into top-class sports. The exercise program developed in the project is to be integrated into the training routine of top athletes. Additional perturbations will be used to vary exercises and induce neuromuscular adaptations. The primary goal of the study is to test the feasibility and acceptance of such a concept in high performance rowing athletes. Secondarily, the effects of such a training shall be recorded by means of an entry and exit diagnostics.

Participants will be athletes in the men's (Pre n=20, Post n=16 - dropout during the qualification phase) and women's (n=10/8) Olympic boat classes. The training intervention is planned to last 12 weeks. The main target criteria will be recorded by an evaluation questionnaire. In addition, there will be a continuous monitoring of the training in connection with a systematic logging of the training content, which in turn will allow the feasibility to be tracked. An analytical consideration of this can be found above all in the final report of the area of
"Transfer" of the research project took place.

The following contents are planned for the longitudinal study (pre/postdiagnostic):

- Isometric strength measurement of the trunk muscles (flexion / extension)
- Postural stability in single-leg stance (a.o. COP)
- Complex sport motor performance (jumping height CMJ)
- Mobility extent (Medi Mouse)
- Back pain prevalence questionnaires /identification of risk factors (von Korff).

Start of the research project: end of April 2018Duration:approx. months3

4. The applicants already have an ethics vote on a comparable research project.

no Yes xIf yes , please provide the following information: Title of research project:

"Evaluation and comparison of an established preventive training program and a developed training [...] in the context of university sports at the Ruhr-University Bochum.""

Evaluation of a developed training program with the focus [...] in the context of the health course AOK RanRücken in cooperation with the AOK Gesundheitskasse".

ethics committee involved:

Ethics Committee of the Faculty of Sports Science (EKS) of the Ruhr University Bochum

Ethics vote file number and date:10/27/2017; 18.12.2017

Checklist and supplementary information on the research project

If "no", supplementary information (see section 9) is mandatory; if "yes", it is optional. In cases of doubt, "no" must be marked with a cross.

Please note the double negative in the statements and9,16.,17.26.!

5. Information to the subjects participating in the research project.

	Facts	yes	no
(1)	An explanation of the general aims of the examination is given.	x	☐
(2)	There is an explanation of the scientific importance of the study, which justifies the effort.	x	☐
(3)	Information about the duration of the examination is provided.	x	☐
(4)	Information is provided on the stresses and risks associated with the examination procedures used and on insurance aspects.	x	☐
(5)	There is a clarification of remuneration and other commitments to the pro-band.	x	☐

(6)	Clarification is provided regarding the voluntary nature of participation.	x	☐
(7)	You will be informed about the possibility of withdrawing from the course at any time and without consequences.	x	☐
(8)	Information is provided on the security of data storage and analysis (anonymization/pseudonymization, who has access to the data).	x	☐
(9)	There is **no** intentional deception of participants (e.g., incomplete or incorrect information about study objectives and procedures, mani- pulized feedback about subject performance).	x	☐
(10)	In the event of intentional deception, the patient will be fully informed of the true aims of the investigation after the end of the test.	☐	☐
(11)	The information is written in a generally understandable way without technical vocabulary and other foreign words.	x	☐
(12)	Feedback of individual examination results to the persons under examination takes place.	x	☐

6. Voluntary participation and vulnerable collectives

	Facts	yes	no
(13)	The voluntary nature of participation is assured.	x	☐
(14)	Only persons capable of giving consent (legally capable adults) are examined or, in the case of examination of persons incapable of giving consent, the consent of their legal representatives (e.g. parents, legal guardian) is obtained.	x	☐
(15)	Only persons who are not subject to a special vulnerable group (e.g., persons with health impairments, children/youth, elderly).	x	☐

7. General conditions of the research project and stress of the test subjects

	Facts	yes	no
(16)	In the context of the research project, the subjects are **not** subjected to any particular physical stress (e.g., by blood sampling, by drug or placebo administration, by invasive measurements, unfamiliar environmental conditions such as hypoxia, subjective exertion perception "very, very strenuous" on the Borg scale, exhaustion test, very high degree of fatigue).	☐	x
(17)	In the context of the research project, the test subjects are **not subject to** any particular psy- chical stresses (e.g., duration of activity, aversive stimuli, negative experiences).	x	☐
(18)	In the case of a special stress in the sense of points 16 and 17, the test subjects are intensively supervised during and after the examination, if necessary.	x	☐
(19)	Subjects do not disclose confidential information or, if such information is collected, were informed of it before signing the consent form.	x	☐
(20)	The persons entrusted with performing the examinations are instructed in detail regarding the procedure and the risks of the methods used (e.g. capillary blood collection).	x	☐
(21)	The applicant consents to the recording of adverse events (e.g., injury or death).<br>The researcher is required to report any adverse events or side effects to the ethics committee in the course of or after completion of the research project.	x	☐

8. Privacy

	Facts	yes	no
(22)	The data is completely anonymized, so that no assignment of the data to persons is possible, or pseudonymized (storage of the data with a personal code, data and names are stored in separate files).	x	☐
(23)	It is ensured that only persons with a confidentiality obligation have access to the personal data (e.g. storage in a locked cabinet, password-protected computer file).	x	☐
(24)	The subjects are informed that they can request the deletion of their data at any time.	x	☐
(25)	The deletion of personal data after the expiry of the statutory retention period is ensured.	x	☐

(26)	There are **no** video or audio recordings or other behavioral registri-. The test is not intended to include any features that could make it possible for third parties to unambiguously identify the test subject.	☐	x

9. Supplementary information on the research project (please insert no.)

Regarding item (16):

During the training sessions, there may be a subjectively high feeling of exertion. However, the load will correspond to the previous training dosage and thus will not represent a higher risk.

Re item (26):

If necessary, picture/sound recordings are made for non-scientific purposes (public relations). However, these require the consent of those responsible and the test persons, as well as a written declaration of consent stating the specific purpose.

Re items (16), (17):

A. Sports science/sports-specific examination procedures/experimental setups/tests.

☐	Actigraphy (e.g. sleep behavior)	☐	Endurance testing
☐	Observations (e.g. lessons); if necessary with video recording	☐	Biofeedback (e.g. skin conductivity, respiratory flow, pulse, temperature)
☐	Bicycle ergometry	☐	Field level test
x	Questionnaires (paper-pencil, online)	☐	Interview; with audio recording if necessary
x	Coordination test	x	Force testing methods (e.g. isometric/dynamic maximum force)
☐	Treadmill test	☐	Motor test
☐	PC-based tests (e.g. attentiveness, reaction time)	☐	Regeneration
x	Sports/medical history	☐	Sprint test (e.g. linear sprint, change of direction sprint, repeat sprint)
x	Jump tests(e.g. CMJ, Drop Jump, Squat Jump, Repeated Jumps)	☐	Speed test procedure
x	Training	☐	Wingate test
☐	________________________	☐	__________________________

B. **Measurement/detection/sensor/measurement condition**

- **extrinsic**
- **passive**
- **non-invasive**

x	Anthropometry	☐	Accelerometer
☐	Bioimpedance	☐	Pressure sensor technology (biomechanics)
☐	ECG	☐	Goniometer
☐	HF	☐	HFV
☐	Contact switch	☐	Force transducer
x	Force plate	☐	LAVEG
☐	Light barrier measurement	☐	Medical history
☐	Muscular function diagnostics	☐	Near Infrared Spectroscopy
☐	Surfaces EMG	☐	Psychometrics
☐	Spirometry	☐	Ultrasound
☐	Video Analysis/High Speed Cam	☐	Time/speed measurement
x	Medi Mouse	☐	______________________________

C. **Measurement/detection/sensor/measurement condition**

- **intrinsic**
- **active**
- **invasive**
- **forced**

☐ Biopsy, removal of tissue	☐ Cervicomedullary stimulation
☐ Diet, fasting	☐ Blood pressure measurement
☐ Administration of medication	☐ Administration of food supplements
☐ Administration of placebos	☐ Hypoxia
☐ Isokinetics	☐ Capillary blood collection
☐ Muscle stimulation	☐ Needle/Finewire EMG
☐ Nerve Stimulation	☐ Posturomed
☐ Saliva examination	☐ Tensiomyography
☐ Transcranial magnetic stimulation	☐ Urinalysis
☐ Venous blood sampling	
☐ ____________________	☐ ____________________

10. Attachments

xWritten information to the subject on the conduct of the studies and on the necessity and risks of the procedures used.

xExplanation of consent by the test person or legal representative.

An application for this research project has not been submitted to any other ethics committee for review.

I certify that all information in this application is accurate to the best of my knowledge.

Bochum21 .03.2018

Place, date

"Feasibility and acceptance of a neuromuscular training concept in elite rowing athletes."

CONSENT FORM

Name, first name:..
Date of birth:..

I agree to participate in the scientific study to evaluate the feasibility of the neuromuscular training concept. I have read and understood all information completely. Any questions that arose were answered in an understandable manner and to my satisfaction.
I have had sufficient time to make up my mind. However, I reserve the right to terminate my voluntary participation at any time. I am aware that I can withdraw my consent to participate in the study at any time and without giving reasons, without incurring any disadvantages. I agree to the collection and processing of data regarding the effectiveness of the training program.

Privacy
The recording and analysis of this data is done pseudonymously in the teaching and research department of sports medicine and sports nutrition at the Ruhr University Bochum, using a number and without giving my name. There exists a coding list on paper, which connects my name with this number. This coding list is only accessible to the experimental management and the project leader, which means that only these persons can link the collected data with my name. After the data analysis is completed, the coding list is deleted. My data are then anonymized. This means that it is no longer possible for anyone to associate the collected data with my name. I am aware that I can revoke my consent to the retention or storage of this data without incurring any disadvantages. I have been informed that I can request deletion of all my data at any time. However, if the coding list has already been deleted, my data record can no longer be identified and therefore cannot be deleted. My data will then be anonymized. I agree that my anonymized data can be further used for research purposes and remain stored for at least 10 years.

☐ Yes, I will participate.

☐ No, I do not participate.

Bochum, the: __________ Signature of subject: ____________________________

Signature Project Staff:

Robin Schäfer (Coordination)
robin.schaefer@rub.de, Tel.: / 0234- 3222080

RUHR-UNIVERSITY BOCHUM |44780 Bochum | Germany

FACULTY OF SPORTS SCIENCE

Department of Sports Medicine
and Sports Nutrition Health
Campus North 10
44801 Bochum

PROF. DR. PETRA PLATEN
Fon +49 (0)234 32-24099
Fax +49 (0)234 32-14323
petra.platen@rub.de

Information for test persons

"Feasibility and acceptability of a neuromuscular training concept in elite rowing athletes."

Dear Sir or Madam,

With this cover letter we would like to inform you about the background and the procedure of the above mentioned study. If you have any questions, please do not hesitate to contact us.

Background and aim of the study

The topic of "back pain" plays a significant role in the general population. According to the Robert Koch Institute (2012), the cut-off date prevalence (occurrence of back pain on any given day) of back pain in Germany is between 32% and 49% and the lifetime prevalence (back pain at least once in a lifetime) is between 74% and 85%. In this context, nonspecific back pain in particular poses a significant problem, as current medical knowledge does not allow for a clear attribution of cause, and thus there is no targeted treatment option. As studies show, this problem also exists in competitive sports.
For this reason, the Federal Institute for Sport Science (BISp) launched the "RAN RÜCKEN" project. A total of scientific13 partners from sports medicine, medicine, psychosocial medicine, sports psychology, health sociology and training science are working together throughout Germany to optimize the diagnosis, prevention and therapy of back pain in society as a whole and in elite sports. In the medium term, the findings should lead to the development of preventive measures for back pain in society as a whole and in elite sports.
In this framework, it will be examined whether and how a transfer of the exercise concept to improve the activation of the trunk muscles is feasible. For optimal trunk stability, certain activation patterns of the trunk musculature must be given in order to prevent damage to the trunk.

to prevent pain. Thus, through improved control of the musculature, trunk stability should also be improved and pain reduced or prevented.

Procedure of the study

Your strength training sessions will be accompanied over a period of twelve weeks. Essentially, your previous training content will be taken up and modified according to the training concept of the research project. During the training phase, the strength training will be supervised by our sports scientists. In addition, the training units will be systematically recorded. Your assistance is required for this.

Tests are performed before and after the training period to check the training effects. Parameters on the isometric maximum strength of the abdominal and back muscles, the extent of mobility of the spine, balance and complex sports motor performance are recorded. In addition, various questionnaires are used. In detail, the tests are as follows:

Isometric maximum force measurement: With the lower body fixed, try to exert maximum force forward (trunk flexion - abdominal muscles) and backward (trunk extension - back muscles) for 5s. The maximum force value can clarify deficits in the abdominal/back muscles or in the ratio of these.

Mobility test: The so-called Medi Mouse is used to record its surface (curvature of the spine) by tracing the back. This is recorded in the positions of lateral tilt (lateral flexion), trunk flexion (flexion) and trunk extension (extension).

Balance: 30-second tests in a single-leg stance on a force plate can be used to record parameters of balance regulation

Complex sport motor performance: As a performance parameter, we determine the jump height during the counter-movement jump (jump with a lunge movement) under standardized conditions.

The questionnaires used will include the following content:

• Medical history (e.g. age, body weight, height)
• Questioning about back pain (e.g. localization, frequency of occurrence, duration, intensity)
• Survey on the management of pain
• Survey on occupational stress
• Survey on activity and leisure/sport behavior
• Survey on the acceptance of the training program
• Feasibility survey
• Survey on the subjective evaluation of the training program

Field-tested questionnaires are used for this purpose.

There is a risk of injury for you (e.g. due to fall injuries during the exercises), which will not exceed the risk of your usual training. Participation in the measurements is also not expected to increase the risk. The respective test situations will be practiced with you in detail and always performed under guidance and supervision. There is no separate insurance cover provided by the study.

Benefit

This study is intended to test the feasibility of the training concept. The results may lead to recommendations for transfer to elite sports. Furthermore, individual exercise forms of the concept that have proven to be practicable can be incorporated into the existing canon of sport-specific exercises that have been created as part of the overall research project. You will receive a personalized, structured training program, as well as feedback on your personal test results. We are also happy to provide training recommendations after the study is completed and offer a final consultation. There is no compensation for participation in the study.

Control group

After the initial diagnostics, you will be informed in a personal interview whether you are in the control group or whether you are participating in the intervention. If you are in the control group, you will continue your training as usual. Random checks of your training sessions will be performed to assess your training volume (in addition to the questionnaire content). After the retest (12 weeks), you will receive a training introduction to the program you have completed. Of course, you will also receive an individual evaluation of your test.

Data protection and voluntariness

When handling your data, the provisions of the Data Protection Act are observed. The recording and evaluation of this data is carried out pseudonymously at the Chair of Sports Medicine and Sports Nutrition at the Ruhr University Bochum using a number and without giving your name. There is a coding list on paper that links your name to this number. This coding list is only accessible to the investigator, which means that only this person can associate the collected data with your name. After the data analysis is completed, the coding list is deleted. Your data are then anonymized. This means that it is no longer possible for anyone to associate the collected data with your name. You can revoke your consent to the retention or storage of this data at any time without incurring any disadvantages. You can request deletion of all your data at any time. However, if the coding list has already been deleted, your data record can no longer be identified and therefore cannot be deleted. Your data is then anonymized. Your anonymized data will be further used for research purposes and will remain 10stored for at least years.

You retain the right to terminate your voluntary participation at any time. You may withdraw your consent to participate in the study at any time without giving reasons and without incurring any disadvantages.

Thank you very much for your time and

assistance! Sincerely yours,

Robin Schäfer (Study
Coordinator)
