# Supplementary material for: Perturbation-based trunk stabilization training in elite rowers: A pilot study
Source: PLoS One. 2022 May 19;17(5):e0268699. doi: 10.1371/journal.pone.0268699 (PMC9119454; doi:10.1371/journal.pone.0268699)
Supplement: S5 File — (PDF) [file pone.0268699.s005.pdf]

RUHR-UNIVERSITÄT BOCHUM | Faku tät für Sportw ssenschaft|
44780 Bochum | Germany

Herr Rob n Schäfer

Lehr- und Forschungsbere ch: Sportmed z n und Sporternährung
Fakultät für Sportw ssenschaft

Ruhr-Un vers tät Bochum

44780 Bochum

Fakultät für Sportwissenschaft
Ethikkommission
Der Vorsitzende
Gebäude SW
Gesundhe tscampus Nord 10
44801 Bochum

[redacted]

Datum
07 05 2018

Sehr geehrter Herr Schäfer,

die Ethikkommission der Fakultät für Sportwissenschaft hat Ihren am 29.03.2018 eingereichten Antrag zu dem Vorhaben mit dem Titel
„Machbarkeit und Akzeptanz eines neuromuskulären Trainingskonzeptes bei Spitzensportlern im Rudern"
zwischenzeitlich geprüft.

Die Kommission hält Ihr Vorhaben für ethisch unbedenklich.

Mit freundlichen Grüßen

[redacted]
Vorsitzender der Ethikkommission der Fakultät für Sportwissenschaft

Antrag auf Stellungnahme der Ethikkommission der Fakultät für Sportwissenschaft (EKS) der Ruhr-Universität Bochum

1. Titel des Forschungsvorhabens

„Machbarkeit und Akzeptanz eines neuromuskulären Trainingskonzeptes bei Spitzensportlern im Rudern“

2. Name und Kontaktdaten der antragstellenden Person:

Name, Vorname: Schäfer, Robin Dienstbez.: wissenschaftlicher Mitarbeiter

Lehr- und Forschungsbereich: Sportmedizin und Sporternährung

E-Mail-Adresse: robin.schaefer@rub.de

Telefonnummer: 0234 / 32 – 22080

3. Kurze Zusammenfassung des Forschungsvorhabens

Ziele, Kollektive, methodisches Vorgehen; max. 250 Wörter

Im Rahmen des langjährigen Forschungsprojektes „Ran Rücken“ sollen die erarbeiteten Konzepte des neuromuskulären Trainings in die Allgemeinbevölkerung, sowie in den Spitzensport transferiert werden. Das im Projekt entstandene Übungsprogramm soll in den Trainingsablauf von Spitzenathleten integriert werden. Durch zusätzliche Perturbationen (Störreize) sollen Übungen variiert und neuromuskuläre Adaptationen hervorgerufen werden. Das Primärziel der Studie ist die Überprüfung der Machbarkeit und der Akzeptanz eines solchen Konzeptes bei Hochleistungssportlern der Sportart Rudern. Sekundär sollen die Effekte eines solchen Trainings durch eine Ein- und Ausgangsdiagnostik erfasst werden.

Teilnehmer sind die Athleten der olympischen Bootsklassen der Männer (Pre n=20, Post n=16 – Dropout während der Qualifikationsphase) und Frauen (n=10/8). Die Trainingsintervention soll 12 Wochen dauern. Die Hauptzielkriterien werden durch einen Evaluationsfragebogen erfasst. Zudem erfolgt eine stetige Betreuung des Trainings im Zusammenhang mit einer systematischen Protokollierung der Trainingsinhalte, wodurch wiederum die Machbarkeit nachvollzogen werden kann. Eine analytische Betrachtung dessen findet vor allem im Abschlussbericht des Bereiches „Transfer“ des Forschungsprojektes statt.

Für die Längsschnittuntersuchung (Prä-/Postdiagnostik) sind folgende Inhalte geplant:

- Isometrische Kraftmessung der Rumpfmuskulatur (Flexion / Extension)
- Posturale Stabilität im Einbeinstand (u.A. COP)
- Komplexe sportmotorische Leistungsfähigkeit (Sprunghöhe CMJ)
- Beweglichkeitsausmaß (Medi Mouse)
- Fragebögen zur Rückenschmerzprävalenz /Ermittlung von Risikofaktoren (von Korff)

Beginn des Forschungsvorhabens: Ende April 2018 Dauer: ca. 3 Monate

4. Es liegt den Antragstellern bereits ein Ethikvotum zu einem vergleichbaren Forschungsvorhaben vor.

nein ☐ ja x Wenn ja, bitte folgende Angaben machen:
Titel des Forschungsvorhabens:

„Evaluation und Vergleich eines etablierten präventiven Trainingsprogrammes und einem entwickelten Training [...] im Rahmen des Hochschulsports der Ruhr-Universität Bochum““

Evaluation eines entwickelten Trainingsprogrammes mit den Schwerpunkten […] im Rahmen des Gesundheitskurses AOK RanRücken in Kooperation mit der AOK Gesundheitskasse“

beteiligte Ethikkommission:

Ethikkomission der Fakultät für Sportwissenschaft (EKS) der Ruhr-Universität Bochum

Aktenzeichen und Datum des Ethikvotums:27.10.2017; 18.12.2017

Checkliste und ergänzende Informationen zum Forschungsvorhaben

Bei „nein“ ist eine ergänzende Information (s. Abschnitt 9) obligatorisch, bei „ja“ fakultativ. In Zweifelsfällen ist „nein“ anzukreuzen.

Bitte die doppelte Verneinung in den Aussagen 9, 16., 17. und 26. beachten!

5. Informationen an die Versuchspersonen, die am Forschungsvorhaben teilnehmen

	Sachverhalt	ja	nein
(1)	Es erfolgt eine Aufklärung über die allgemeinen Untersuchungsziele.	x	☐
(2)	Es erfolgt eine Aufklärung über die wissenschaftliche Bedeutung der Studie, die den Aufwand rechtfertigt.	x	☐
(3)	Es erfolgt eine Aufklärung über die Dauer der Untersuchung.	x	☐
(4)	Es erfolgt eine Aufklärung über Belastungen und Risiken durch eingesetzte Untersuchungsverfahren sowie über Versicherungsaspekte.	x	☐
(5)	Es erfolgt eine Aufklärung über Vergütungen und andere Zusagen an die Proband/innen.	x	☐

(6)	Es erfolgt eine Aufklärung über die Freiwilligkeit der Teilnahme.	x	
(7)	Es erfolgt eine Aufklärung über die jederzeitige und folgenlose Rücktrittsmöglichkeit von der Teilnahme.	x	
(8)	Es erfolgt eine Aufklärung über die Sicherheit der Aufbewahrung und Auswertung der Daten (Anonymisierung/Pseudonymisierung, wer Zugriff auf die Daten hat).	x	
(9)	Es findet **keine** absichtliche Täuschung der Teilnehmer statt (z. B. unvollständige oder falsche Information über Untersuchungsziele und -verfahren, manipulierte Rückmeldungen über Proband/innen-Leistungen).	x	
(10)	Es wird im Falle einer absichtlichen Täuschung nach Beendigung des Versuchs umfassend über die wahren Untersuchungsziele aufgeklärt.		
(11)	Die Informationen sind allgemeinverständlich ohne Fachvokabular und andere Fremdwörter abgefasst.	x	
(12)	Eine Rückmeldung von individuellen Untersuchungsergebnissen an die untersuchten Personen findet statt.	x	

6. Freiwilligkeit der Teilnahme und vulnerable Kollektive

	Sachverhalt	ja	nein
(13)	Die Freiwilligkeit der Teilnahme ist gesichert.	x	
(14)	Es werden nur einwilligungsfähige Personen untersucht (rechtsfähige Erwachsene) oder es wird im Falle der Untersuchung nicht einwilligungsfähiger Personen die Einwilligung der gesetzlichen Vertreter (z. B. Eltern, gesetzliche Betreuungsperson) eingeholt.	x	
(15)	Es werden an der Untersuchung nur Personen teilnehmen, die keiner besonders verletzlichen Gruppe angehören (z. B. gesundheitlich eingeschränkte Personen, Kinder/Jugendliche, ältere Menschen).	x	

7. Rahmenbedingungen des Forschungsvorhabens und Beanspruchung der Versuchspersonen

	Sachverhalt	ja	nein
(16)	Im Rahmen des Forschungsvorhabens werden die Versuchspersonen physisch **nicht** besonders beansprucht (z. B. durch Entnahme von Blut, durch Medikamenten- oder Placebogaben, durch invasive Messungen, ungewohnte Umweltbedingungen wie Hypoxie, subjektives Anstrengungsempfinden „sehr, sehr anstrengend" auf der Borg-Skala, Ausbelastungstest, sehr hoher Ermüdungsgrad).	☐	x
(17)	Im Rahmen des Forschungsvorhabens werden die Versuchspersonen psychisch **nicht** besonders beansprucht (z. B. durch Tätigkeitsdauer, aversive Reize, negative Erfahrungen).	x	☐
(18)	Im Fall einer besonderen Beanspruchung i. S. der Punkte 16 und 17 werden die Versuchspersonen während und nach der Untersuchung bei Bedarf intensiv betreut.	x	☐
(19)	Die Versuchspersonen geben keine vertraulichen Informationen preis oder wurden – falls solche Informationen erfasst werden – vor Unterzeichnung der Einwilligungserklärung darüber informiert.	x	☐
(20)	Die mit der Durchführung der Untersuchungen betrauten Personen sind bzgl. des Ablaufs und der Risiken der angewendeten Verfahren ausführlich instruiert (z. B. Kapillarblutentnahme).	x	☐
(21)	Die antragstellende Person willigt ein, unerwünschte Ereignisse (z. B. Verletzungen, Nebenwirkungen) im Verlauf bzw. nach Beendigung des Forschungsvorhabens an die Ethikkommission zu melden.	x	☐

8. Datenschutz

	Sachverhalt	ja	nein
(22)	Die Daten werden vollständig anonymisiert, so dass keine Zuordnung der Daten zu Personen möglich ist, oder pseudonymisiert (Speicherung der Daten mit einem Personen-Code, Daten und Namen werden in getrennten Dateien gespeichert).	x	☐
(23)	Es ist sichergestellt, dass nur schweigeverpflichtete Personen einen Zugriff zu den persönlichen Daten haben (z. B. Aufbewahrung in verschlossenem Schrank, passwortgeschützte Computerdatei).	x	☐
(24)	Die Versuchspersonen werden darauf hingewiesen, jederzeit die Löschung ihrer Daten verlangen zu können.	x	☐
(25)	Die Löschung personenbezogener Daten nach Ablauf der gesetzlichen Aufbewahrungsfrist ist gesichert.	x	☐

(26)	Es sind **keine** Video- oder Tonaufnahmen oder andere Verhaltens- Registrierungen vorgesehen, welche eine eindeutige Identifizierung der Versuchsperson durch Dritte möglich machen könnten.	☐	x

9. Ergänzende Informationen zum Forschungsvorhaben (bitte Nr. einfügen)

Zu Punkt (16):

Während der Trainingseinheiten kann es zu subjektiv hohem Anstrengungsempfinden kommen. Die Belastung wird allerdings der bisherigen Trainingsdosierung entsprechen und somit kein höheres Risiko darstellen.

Zu Punkt (26):

Ggf. werden Bild-/Tonaufnahmen zu nicht wissenschaftlichen Zwecken (Öffentlichkeitsarbeit) angefertigt. Diese bedürfen allerdings der Zustimmung der Verantwortlichen und der Probanden, als auch eine schriftliche Einverständniserklärung aus welcher der konkrete Zweck hervogeht.

Zu den Punkten (16), (17):

A. Sportwissenschaftliche/sportspezifische Untersuchungsverfahren/Versuchsanordnungen/Tests

☐	Aktigraphie (z. B. Schlafverhalten)	☐	Ausdauertestverfahren
☐	Beobachtungen (z. B. Unterricht); ggf. mit Videoaufzeichnung	☐	Biofeedback (z. B. Hautleitfähigkeit, Atemfluss, Puls, Temperatur)
☐	Fahrradergometrie	☐	Feldstufentest
x	Fragebögen (paper-pencil, online)	☐	Interview; ggf. mit Audioaufzeichnung
x	Koordinationstest	x	Krafttestverfahren (z. B. isometrische/dynamische Maximalkraft)
☐	Laufbandtest	☐	Motorischer Test
☐	PC-gestützte Tests (z. B. Aufmerksamkeit, Reaktionszeit)	☐	Regeneration
x	Sportliche/medizinische Anamnese	☐	Sprinttest (z. B. Linearsprint, Richtungswechselsprint, Wiederholungssprint)
x	Sprungtests(z. B. CMJ, Drop Jump, Squat Jump, Repeated Jumps)	☐	Schnelligkeitstestverfahren
x	Training	☐	Wingate Test
☐	____________________	☐	____________________

B. **Messung/Erfassung/Sensor/Messbedingung**
- **extrinsisch**
- **passiv**
- **non-invasiv**

x	Anthropometrie	☐	Beschleunigungsaufnehmer
☐	Bioimpedanz	☐	Drucksensorik (Biomechanik)
☐	EKG	☐	Goniometer
☐	HF	☐	HFV
☐	Kontaktschalter	☐	Kraftaufnehmer
x	Kraftmessplatte	☐	LAVEG
☐	Lichtschrankenmessung	☐	Medizinische Anamnese
☐	Muskuläre Funktionsdiagnostik	☐	Nahinfrarotspektroskopie
☐	Oberflächen EMG	☐	Psychometrie
☐	Spirometrie	☐	Ultraschall
☐	Videoanalyse/High-Speed-Cam	☐	Zeit-/Geschwindigkeitsmessung
x	Medi Mouse	☐	______________________________

C. **Messung/Erfassung/Sensor/Messbedingung**
- **intrinsisch**
- **aktiv**
- **invasiv**
- **zwangsbedingt**

☐ Biopsie, Entnahme von Gewebe	☐ Cervicomedullare Stimulation
☐ Diät, Fasten	☐ Blutdruckmessung
☐ Gabe von Medikamenten	☐ Gabe von Nahrungsergänzungsmitteln
☐ Gabe von Placebos	☐ Hypoxie
☐ Isokinetik	☐ Kapillarblutentnahme
☐ Muskelstimulation	☐ Nadel/Finewire-EMG
☐ Nervstimulation	☐ Posturomed
☐ Speicheluntersuchung	☐ Tensiomyografie
☐ Transkranielle Magnetstimulation	☐ Urinuntersuchung
☐ Venöse Blutentnahme	
☐ ______________________________	☐ ______________________________

10. Anlagen

x Schriftliche Aufklärung der Versuchsperson zur Durchführung der Untersuchungen sowie zur Notwendigkeit und den Risiken der eingesetzten Verfahren

x Einwilligungserklärung der Versuchsperson bzw. der gesetzlichen Vertretung

Ein Antrag zu diesem Forschungsvorhaben wurde nicht bei einer anderen Ethikkommission zur Begutachtung eingereicht.

Ich bestätige, dass alle Angaben in diesem Antrag nach bestem Wissen zutreffend sind.

Bochum 21.03.2018

Ort, Datum

„Machbarkeit und Akzeptanz eines neuromuskulären Trainingskonzeptes bei Spitzensportlern im Rudern“

EINVERSTÄNDNISERKLÄRUNG

Name, Vorname:………………………………...
Geburtsdatum:…………………………………………………..………………………

Ich erkläre mich bereit, an der wissenschaftlichen Studie zur Evaluierung der Durchführbarkeit des neuromuskulären Trainingskonzeptes teilzunehmen. Ich habe alle Informationen vollständig gelesen und verstanden. Aufgetretene Fragen wurden verständlich und zu meiner Zufriedenheit beantwortet.
Ich hatte ausreichend Zeit, mich zu entscheiden. Ich behalte mir jedoch das Recht vor, meine freiwillige Mitwirkung jederzeit zu beenden. Mir ist bekannt, dass ich jederzeit und ohne Angabe von Gründen meine Einwilligung zur Teilnahme an der Studie zurückziehen kann, ohne dass mir daraus Nachteile entstehen. Mit der Erhebung und Verarbeitung der Daten bezüglich der Effektivität des Trainingsprogramms bin ich einverstanden.

Datenschutz
Die Aufzeichnung und Auswertung dieser Daten erfolgt pseudonymisiert im Lehr- und Forschungsbereich Sportmedizin und Sporternährung der Ruhr-Universität Bochum, unter Verwendung einer Nummer und ohne Angabe meines Namens. Es existiert eine Kodierliste auf Papier, die meinen Namen mit dieser Nummer verbindet. Diese Kodierliste ist nur der Versuchsleitung und dem Projektleiter zugänglich, das heißt, nur diese Personen können die erhobenen Daten mit meinem Namen in Verbindung bringen. Nach Abschluss der Datenauswertung wird die Kodierliste gelöscht. Meine Daten sind dann anonymisiert. Damit ist es niemandem mehr möglich, die erhobenen Daten mit meinem Namen in Verbindung zu bringen. Mir ist bekannt, dass ich mein Einverständnis zur Aufbewahrung bzw. Speicherung dieser Daten widerrufen kann, ohne dass mir daraus Nachteile entstehen. Ich bin darüber informiert worden, dass ich jederzeit eine Löschung all meiner Daten verlangen kann. Wenn allerdings die Kodierliste bereits gelöscht ist, kann mein Datensatz nicht mehr identifiziert und somit auch nicht mehr gelöscht werden. Meine Daten sind dann anonymisiert. Ich bin einverstanden, dass meine anonymisierten Daten zu Forschungszwecken weiterverwendet werden können und mindestens 10 Jahre gespeichert bleiben.

☐ Ja, ich nehme teil.

☐ Nein, ich nehme nicht teil.

Bochum, den: _________ Unterschrift Proband:____________________________

Unterschrift Projektmitarbeiter:_________________________

Robin Schäfer (Koordination)
robin.schaefer@rub.de, Tel.: 0234 / 32 - 22080

RUHR-UNIVERSITÄT BOCHUM | 44780 Bochum | Germany

FAKULTÄT FÜR SPORTWISSENSCHAFT

Lehr- und Forschungsbereich
Sportmedizin und Sporternährung
Gesundheitscampus Nord 10
44801 Bochum

PROF. DR. PETRA PLATEN
Fon +49 (0)234 32-24099
Fax +49 (0)234 32-14323
petra.platen@rub.de

Information für Probandinnen und Probanden

„Machbarkeit und Akzeptanz eines neuromuskulären Trainingskonzeptes bei Spitzensportlern im Rudern."

Sehr geehrte Damen und Herren,

mit diesem Anschreiben möchten wir Sie über den Hintergrund und den Ablauf der oben genannten Studie informieren. Bei Rückfragen stehen wir Ihnen jederzeit gerne zur Verfügung.

Hintergrund und Ziel der Studie
Das Thema „Rückenschmerz" spielt in der Allgemeinbevölkerung eine bedeutende Rolle. Nach Angaben des Robert-Koch-Instituts (2012) liegt die Stichtagprävalenz (Vorkommen des Rückenschmerzes an einem beliebigen Tag) von Rückenschmerzen in Deutschland zwischen 32% und 49% und die Lebenszeitprävalenz (mindestens einmal Rückenschmerzen im Leben) zwischen 74% und 85%. Insbesondere der unspezifische Rückenschmerz stellt in diesem Zusammenhang ein erhebliches Problem dar, da mit den heutigen medizinischen Kenntnissen keine eindeutige Ursachenzuschreibung erfolgen kann und somit keine gezielte Behandlungsmöglichkeit besteht. Dieses Problem besteht, wie Untersuchungen zeigen, auch im Leistungssport.
Daher wurde vom Bundesinstitut für Sportwissenschaft (BISp) das Projekt „RAN RÜCKEN" ins Leben gerufen. Insgesamt 13 wissenschaftliche Partner aus der Sportmedizin, Medizin, psychosozialen Medizin, Sportpsychologie, Gesundheitssoziologie und Trainingswissenschaft arbeiten deutschlandweit gemeinsam an der Optimierung der Diagnose, Prävention und Therapie von Rückenschmerzen in der Gesamtgesellschaft und im Spitzensport. Die Erkenntnisse sollen mittelfristig zur Entwicklung präventiver Maßnahmen von Rückenschmerzen in der Gesellschaft und im Spitzensport führen.
In diesem Rahmen soll überprüft werden, ob und wie ein Transfer des Übungskonzeptes zur Verbesserung der Aktivierung der Rumpfmuskulatur durchführbar ist. Für eine optimale Rumpfstabilität müssen gewisse Aktivierungsmuster der Rumpfmuskulatur gegeben sein, um Schäden

vorzubeugen. Somit soll durch eine verbesserte Ansteuerung der Muskulatur auch die Rumpfstabilität verbessert und Schmerzen reduziert oder verhindert werden.

Ablauf der Studie

Ihre Krafttrainingseinheiten werden über einen Zeitraum von zwölf Wochen begleitet. Im Wesentlichen werden Ihre bisherigen Trainingsinhalte aufgegriffen und nach dem Trainingskonzept des Forschungsprojektes modifiziert. Während der Trainingsphase wird das Krafttraining durch unsere Sportwissenschaftler betreut. Außerdem werden die Trainingseinheiten systematisch protokolliert. Dabei ist Ihre Mithilfe erforderlich.

Zur Überprüfung der Trainingseffekte werden vor und nach der Trainingsperiode Tests durchgeführt. Es werden Parameter zur isometrischen Maximalkraft der Bauch- und Rückenmuskulatur, des Beweglichkeitsausmaßes der Wirbelsäule, des Gleichgewichts und der komplexen sportmotorischen Leistung erfasst. Zudem werden diverse Fragebögen eingesetzt. Im Detail sehen die Tests wie folgt aus:

Isometrische Maximalkraftmessung: Mit fixiertem Unterkörper versuchen Sie für 5s maximale Kraft nach vorne (Rumpfbeuge – Bauchmuskulatur) und nach hinten (Rumpfstreckung – Rückenmuskulatur) auszuüben. Der maximale Kraftwert kann über Defizite der Bauch-/Rückenmuskulatur oder im Verhältnis dieser aufklären.

Beweglichkeitstest: Mit der sogenannten Medi Mouse wird durch Abfahren des Rückens dessen Oberfläche (Krümmung der Wirbelsäule) erfasst. Diese wird in den Positionen der Seitneigung (Lateralflexion), der Rumpfbeuge (Flexion) und Rumpfstreckung (Extension) erfasst

Gleichgewicht: Durch 30-sekündige Tests im Einbeinstand auf einer Kraftmessplatte können Parameter der Gleichgewichtsregulation erfasst werden

Komplexe sportmotorische Leistungsfähigkeit: Als Leistungsparameter ermitteln wir die Sprunghöhe beim Counter-Movement-Jump (Sprung mit Ausholbewegung) unter standardisierten Bedingungen.

Die eingesetzten Fragebögen werden folgende Inhalte umfassen:

- Anamnese (z.B. Alter, Körpergewicht, Körpergröße)
- Befragung zum Rückenschmerz (z.B. Lokalisation, Auftretenshäufigkeit, Dauer, Intensität)
- Befragung zum Umgang mit Schmerzen
- Befragung zur beruflichen Belastung
- Befragung zur Aktivität und zum Freizeit-/Sportverhalten
- Befragung zur Akzeptanz des Trainingsprogrammes
- Befragung zur Machbarkeit
- Befragung zur subjektiven Beurteilung des Trainingsprogrammes

Hierzu werden praxiserprobte Fragebögen eingesetzt.

Es besteht für Sie ein Verletzungsrisiko (z.B. durch Sturzverletzungen während der Übungen), welches das Risiko ihres gewöhnlichen Trainings nicht übersteigen wird. Durch die Teilnahme an den Messungen ist ebenfalls nicht mit einer Zunahme des Risikos zu rechnen. Die jeweiligen Testsituationen werden mit Ihnen ausführlich geübt und immer unter Anleitung und Aufsicht durchgeführt. Ein gesonderter Versicherungsschutz durch die Studie besteht nicht.

Nutzen
Durch diese Studie soll die Durchführbarkeit des Trainingskonzeptes überprüft werden. Aus den Ergebnissen können sich Empfehlungen für den Transfer in den Spitzensport ergeben. Des Weiteren können einzelne Übungsformen des Konzeptes, die sich als praktikabel erwiesen haben, in den bereits bestehenden Kanon sportartspezifischer Übungen, die im Rahmen des gesamten Forschungsprojektes entstanden sind, eingegliedert werden. Sie erhalten ein personalisiertes, strukturiertes Trainingsprogramm, sowie Rückmeldungen über ihre persönlichen Testergebnisse. Gerne geben wir auch Trainingsempfehlungen nach Beendigung der Studie und bieten ein Abschlussgespräch an. Für die Teilnahme an der Studie gibt es keine Vergütung.

Kontrollgruppe
Nach der Eingangsdiagnostik wird Ihnen in einem persönlichen Gespräch mitgeteilt, ob Sie in der Kontrollgruppe sind oder an der Intervention teilnehmen. Sind Sie Proband in der Kontrollgruppe, führen Sie ihr Training wie gewohnt fort. Es werden stichprobenartige Überprüfungen ihrer Trainingseinheiten durchgeführt um ihr Trainingsvolumen (ergänzend zu den Fragebogeninhalten) abzuschätzen. Nach dem Retest (12 Wochen) erhalten Sie eine Trainingseinführung in das durchgeführte Programm. Ebenfalls erhalten Sie natürlich auch eine individuelle Auswertung ihrer Testung.

Datenschutz und Freiwilligkeit
Beim Umgang mit Ihren Daten werden die Bestimmungen des Datenschutzgesetzes beachtet. Die Aufzeichnung und Auswertung dieser Daten erfolgt pseudonymisiert am Lehrstuhl für Sportmedizin und Sporternährung der Ruhr-Universität Bochum unter Verwendung einer Nummer und ohne Angabe Ihres Namens. Es existiert eine Kodierliste auf Papier, die Ihren Namen mit dieser Nummer verbindet. Diese Kodierliste ist nur dem Versuchsleiter zugänglich, das heißt, nur diese Person kann die erhobenen Daten mit Ihrem Namen in Verbindung bringen. Nach Abschluss der Datenauswertung wird die Kodierliste gelöscht. Ihre Daten sind dann anonymisiert. Damit ist es niemandem mehr möglich, die erhobenen Daten mit Ihrem Namen in Verbindung zu bringen. Sie können Ihr Einverständnis zur Aufbewahrung bzw. Speicherung dieser Daten jederzeit widerrufen, ohne dass Ihnen daraus Nachteile entstehen. Sie können jederzeit eine Löschung all Ihrer Daten verlangen. Wenn allerdings die Kodierliste bereits gelöscht ist, kann Ihr Datensatz nicht mehr identifiziert und somit auch nicht mehr gelöscht werden. Ihre Daten sind dann anonymisiert. Ihre anonymisierten Daten werden zu Forschungszwecken weiterverwendet und bleiben mindestens 10 Jahre gespeichert.

Ihnen bleibt das Recht vorbehalten, Ihre freiwillige Mitwirkung jederzeit zu beenden. Sie können jederzeit ohne Angabe von Gründen Ihre Einwilligung zur Teilnahme an der Studie zurückziehen, ohne dass Ihnen Nachteile daraus entstehen.

Vielen Dank für Ihre Zeit und Ihre Mithilfe!

Mit freundlichen Grüßen,

Robin Schäfer
(Studienkoordinator)
