# Supplementary material for: Perturbation-based trunk stabilization training in elite rowers: A pilot study
Source: PLoS One. 2022 May 19;17(5):e0268699. doi: 10.1371/journal.pone.0268699 (PMC9119454; doi:10.1371/journal.pone.0268699)
Supplement: Attachment — Submitted filename: Response to Reviewers.docx [file pone.0268699.s006.docx]

**Rebuttal Letter – Response to Reviewers**

Dear Editor and Reviewers,

thank you for your honest and thorough review of our manuscript. In the following we present our response to all comments raised.

**PONE-D-21-13623**

**Perturbation-based Trunk Stabilization Training in Elite Rowers**

**Journal Requirements:**

When submitting your revision, we need you to address these additional requirements.

1. Please ensure that your manuscript meets PLOS ONE's style requirements, including those for file naming. The PLOS ONE style templates can be found at 
<https://journals.plos.org/plosone/s/file?id=wjVg/PLOSOne_formatting_sample_main_body.pdf> and 
<https://journals.plos.org/plosone/s/file?id=ba62/PLOSOne_formatting_sample_title_authors_affiliations.pdf>

\# changed captions for figures (fig) and tables („.“ Instead of „|“ as separator

2. Thank you for submitting your clinical trial to PLOS ONE and for providing the name of the registry and the registration number. The information in the registry entry suggests that your trial was registered after patient recruitment began. PLOS ONE strongly encourages authors to register all trials before recruiting the first participant in a study.

As per the journal’s editorial policy, please include in the Methods section of your paper:
a) your reasons for your delay in registering this study (after enrolment of participants started);
b) confirmation that all related trials are registered by stating: “The authors confirm that all ongoing and related trials for this drug/intervention are registered

\# done

3. Please note that in order to use the direct billing option the corresponding author must be affiliated with the chosen institute. Please either amend your manuscript to change the affiliation or corresponding author, or email us at plosone@plos.org with a request to remove this option.

\# I am affiliated (RS) – I can’t see a problem here…

4. Please ensure that you refer to Figure 1 in your text as, if accepted, production will need this reference to link the reader to the figure.

\# done

5. We note that Figure 2 in your submission contain copyrighted images. All PLOS content is published under the Creative Commons Attribution License (CC BY 4.0), which means that the manuscript, images, and Supporting Information files will be freely available online, and any third party is permitted to access, download, copy, distribute, and use these materials in any way, even commercially, with proper attribution. For more information, see our copyright guidelines: http://journals.plos.org/plosone/s/licenses-and-copyright.

We require you to either (1) present written permission from the copyright holder to publish these figures specifically under the CC BY 4.0 license, or (2) remove the figures from your submission:

a. You may seek permission from the original copyright holder of Figure 2 to publish the content specifically under the CC BY 4.0 license.

We recommend that you contact the original copyright holder with the Content Permission Form (http://journals.plos.org/plosone/s/file?id=7c09/content-permission-form.pdf) and the following text:
“I request permission for the open-access journal PLOS ONE to publish XXX under the Creative Commons Attribution License (CCAL) CC BY 4.0 (http://creativecommons.org/licenses/by/4.0/). Please be aware that this license allows unrestricted use and distribution, even commercially, by third parties. Please reply and provide explicit written permission to publish XXX under a CC BY license and complete the attached form.”

Please upload the completed Content Permission Form or other proof of granted permissions as an "Other" file with your submission.

In the figure caption of the copyrighted figure, please include the following text: “Reprinted from [ref] under a CC BY license, with permission from [name of publisher], original copyright [original copyright year].”

b. If you are unable to obtain permission from the original copyright holder to publish these figures under the CC BY 4.0 license or if the copyright holder’s requirements are incompatible with the CC BY 4.0 license, please either i) remove the figure or ii) supply a replacement figure that complies with the CC BY 4.0 license. Please check copyright information on all replacement figures and update the figure caption with source information. If applicable, please specify in the figure caption text when a figure is similar but not identical to the original image and is therefore for illustrative purposes only.

\# We hold the copyright for the material presented and present the material here on our website: https://mispex.de/ran-ruecken-sport/ . Also, we have uploaded the figures from the publication into our repository where we store supplementary material. They are stored under the CC BY 4.0 License here: <https://osf.io/d983m/> .

6. Please include captions for your Supporting Information files at the end of your manuscript, and update any in-text citations to match accordingly. Please see our Supporting Information guidelines for more information: <http://journals.plos.org/plosone/s/supporting-information> .

\# replaced the direct links inserted in the manuscript with S1 and S2. Provided the links in the Data accessibility statement

7. We note that Figure 2 includes an image of a participant in the study. 
As per the PLOS ONE policy (http://journals.plos.org/plosone/s/submission-guidelines#loc-human-subjects-research) on papers that include identifying, or potentially identifying, information, the individual(s) or parent(s)/guardian(s) must be informed of the terms of the PLOS open-access (CC-BY) license and provide specific permission for publication of these details under the terms of this license. Please download the Consent Form for Publication in a PLOS Journal (http://journals.plos.org/plosone/s/file?id=8ce6/plos-consent-form-english.pdf). The signed consent form should not be submitted with the manuscript, but should be securely filed in the individual's case notes. Please amend the methods section and ethics statement of the manuscript to explicitly state that the patient/participant has provided consent for publication: “The individual in this manuscript has given written informed consent (as outlined in PLOS consent form) to publish these case details”.

If you are unable to obtain consent from the subject of the photograph, you will need to remove the figure and any other textual identifying information or case descriptions for this individual.

\# written consent was obtained from both athletes

**Reviewers' comments:**

**Reviewer #1:** A two-arm controlled study was conducted to investigate the effects of perturbation-based trunk stabilization training (PTT) on back pain intensity and disability, maximum isometric trunk extension and flexion, jump height and postural sway of single-leg stance in male members of a German rowing team. Less disability was observed in the PTT group than in controls. Similar decreases in pain were observed in both groups.

Minor revisions:
1- In the abstracts, indicate that the study was non-randomized.

\# done (line 17)

2- Prior to applying the ANCOVA, indicate if the distribution of the data checked for normality.

\# added statement for assumption checks (normality and homoscedasticity) – line 193/194 (now 204/205)

3- Since the study was not randomized, provide p-values to compare the baseline characteristics shown in Table 1.

\# IMO, p-values are still not reasonable, as elaborated in the STROBE guideline, section 14 (https://www.ncbi.nlm.nih.gov/pmc/articles/PMC2020496/):

*„Inferential measures such as standard errors and confidence intervals should not be used to describe the variability of characteristics, and significance tests should be avoided in descriptive tables. Also, P values are not an appropriate criterion for selecting which confounders to adjust for in analysis; even small differences in a confounder that has a strong effect on the outcome can be important [144,145].“*

*To guide the reader though, we added the mean difference + confidence intervals.*

4- The standard statistical terminology for “average” is “mean.”

\# changed in lines 201, 136, 126, 14, 157 to mean.

**Reviewer #2:** Dear all
I realize that authors have many journals to consider when they want to publish their work, so I appreciate your interest in PLOS ONE; I am very happy to be able to write in a positive way.
It is evident that you have put a great deal of effort into this project and I want to praise your efforts,
The actual contribution from your study is clear and strong. The manuscript as currently written suggests that it might be suitable for sharing information about this field, and the data that you reported are representative to state with certainty your conclusions.
I should like to thank you for give me an opp ortunity to consider this work for publication. Great paper.
There is an error at introduction section at fourth line, replace low back pain with LBP.
Best Regards

\# thank you for the kind words! We replaced „low back pain“ with LBP at line 36. We also rewrote this sentence based on suggestions by Reviewer #3

**Reviewer #3:** Thank you for the opportunity to review this paper. General Comments:
Unfortunately I think the paper has some challenges. A significant number of these have been eluded to by the authors in the limitations section but they are issues that require addressing before publication.
in particular the study is not as robust as it could because, a) the participants were not randomly allocated to the intervention and control group, b) the study is underpowered c) the power analysis does indicate what the required number would be to potentially reach significance but this was indicated retrospectively d) the results do not show any real meaningful differences between the groups so this does not really add to the body of knowledge in this area. Perhaps the paper could be reworked as a pilot study then the results could be used to inform a more robust design that tests to see if the perturbation training actually is effective. The paper could also do with some review of the written English in places.

\# First of all, thank you for your honest and thorough review and for challenging our paper! As addressed in the paper, we agree with most of your critiques, but we also want to strengthen our standpoint before considering the specific comments raised. From a purely scientific standpoint and in an ideal world we would have addressed all these points before conducting the study (and we certainly argued about these) – but the „dilemma“ we are facing was again recently highlighted by Skorski & Hecksteden (<https://doi.org/10.1123/ijspp.2021-0467>). We examined training effects for the German national team which are deemed to be the best sweep rowers in Germany and also among the best in the world. Skorski & Hecksteden wrote:

*„Currently, the prevailing approach is to justify a suboptimal sample size by the elite status of the participating athletes. Alternatively, athletes with a lower performance level are included. However, the former is beset with the mentioned limitations and the latter questions the transferability to the elite level.“*

Addressing points b) and c) (the power /representativeness problem), we would have to include sub-elite athletes from Germany or elite rowers from other countries. While the latter is not feasible, the former would be statistically spoken more sound (generalizability) but maybe not so representative for the elite athletes. We just don’t have that many elite athletes (winner’s curse). Non-randomization remains a huge critique point (though, baseline values were on average similar in the outcomes, but not in certain characteristics like age and training volume). But you simply can’t go to a top team, change training habits and expect them to split up a team into two training groups. It was hard enough to convince coaches to change training habits in general, while the team is successful.

On point d) I highly disagree. While 5 points disability (on average) may not seem too much in the first place, it is almost in line with findings in the general population and a recent meta-analysis on elite athletes (Thornton et al. (2021) - <http://dx.doi.org/10.1136/bjsports-2020-102723>). We argued in our discussion, that a very small change is still worthwhile and can outperform risks (while the only one seems to be prioritizing other training contents). We found 5 points irrespective of „floor“ effects (some even had negligible pain/disability values – therefore the subgroup analysis). Disability was not very high on baseline assessment. While this may be a critique of our findings as well, better tools for athletes LBP assessment have still to be developed.

We certainly agree that we cannot draw strong conclusions based on the data from this study. Therefore, we used probabilistic language („possibly effective“) to express our uncertainty when writing the conclusion to express our interpretation including. This interpretation is not solely determined by the data presented from this study, but also the evidence from the literature as explained by Amrhein et al. (2018 - <https://doi.org/10.1080/00031305.2018.1543137>). Exercise seems to be effective for LBP in elite athletes, but no superior exercise mode could be identified (Thornton et al. 2021) – similar findings as in the general population, despite the effect sizes being quite smaller. Though, there is a lack of high-quality RCT’s, which our study, unfortunately, can’t fill. But we still think it is worth considering this study as part of the puzzle.

We agree also, that the study could be viewed as a pilot study. For this reason, we included a power- calculation for a study, that could be carried out with elite / sub-elite athletes. We don’t like to include a power analysis in the methods section, because we like to honestly report the sample size determination, which is purely based on feasibility constraints.

Regarding the language: We work out the comments and try to improve the style of the paper. If the revised version could still need revision, we agree to contact native speakers for proofreading, if desired.

**Specific Comments**
Title: I wonder if a better title might be Perturbation based trunk motor control in elite rowers. The main construct of the paper is on motor control rather than stabilisation. Stabilisation is a more challenging term and implies changing an unstable situation. These rowers need to show greater control of trunk flexion as indicated in some of the introduction sections

\# We understand, that „stabilisation“ might be misleading in this context. Though, the term is usually used in the German language as a conglomerate term for describing many kinds of strengthening exercises. In our opinion, motor control might be more misleading, as this is often associated with segmental stabilization via voluntary contractions of deep muscles and similar training procedures. We argue in lines 67-71, that these methods quite differ and nomenclature is ambiguous at its best. Another possibility could be „Perturbation-based sensorimotor training“ which is how it is often called within Germany. Arampatzis et al. (2021) title for a similar study was “Perturbation‐based exercise for prevention of low‐back pain in adolescent athletes”, so an alternative could be “Perturbation‐based exercise for prevention of low‐back pain in elite rowers”. Nevertheless, thank you for your suggestion, but we think the title as it is now is appropriate as well.

**Introduction**

line 34 insert the word the before Consequences

#done

Line 35 insert the word a before breakdown

#done

Line 35 reword for example, rowers missed training to with rowers often missing training. This first paragraph is an example of three changes in English that improve the readability. Please look at other improvements where noted in the paper

\# thank you for outlining these specific changes. I was wondering if there are more specific comments we could use for improvement, that are not part of the revision text here. If so, is there a possibility to receive the document with your annotations?

This whole first section of low back pain in rowing needs to be situated in the terms on the latest consensus statement on LBP and rowing and refer to this as rowing related LBP see Wilson, F., Thornton, J. S., Wilkie, K., Hartvigsen, J., Vinther, A., Ackerman, K. E., Caneiro, J. P., Trease, L., Nugent, F., Gissane, C., McDonnell, S. J., McGregor, A., Newlands, C., & Ardern, C. L. (2021, Mar 8). 2021 consensus statement for preventing and managing low back pain in elite and subelite adult rowers. Br J Sports Med. https://doi.org/10.1136/bjsports-2020-103385 
I think there are also other references that could be used around the incidence and prevalence of RR LBP see
Newlands, C., Reid, D., & Parmar, P. (2015, Jul). The prevalence, incidence and severity of low back pain among international-level rowers. Br J Sports Med, 49(14), 951-956. https://doi.org/10.1136/bjsports-2014-093889 

\# To be honest, I was aware of the paper series in BJSM when submitting the article. Our draft was finished in October 2020 and was submitted in April 2021, and we fell short on citing those papers. Nevertheless, we don’t like to completely change our argumentation post-hoc. Points that are worth addressing are possibly the environmental/educational aspect and the biopsychosocial framework (eg, yellow flags). The former is probably a consequence of our study (the team is using the training principles still today and is probably more aware of LBP in general). On the latter, we again fell short and might be blamable of a (too) narrow biomedical perspective as it is shown in Mescouto et al. (2020 - <https://doi.org/10.1080/09638288.2020.1851783>) and certainly papers from the BJSM series. But we can’t change this post-hoc.

We now refer to the paper for sake of the previous last sentence in the first paragraph. We could cover the points mentioned above in the „practical application“ section to embrace the importance of a holistic approach to LBP rather than relying only on exercise.

Regarding Newlands et al.: I cited this paper in an earlier draft in the first paragraph, but I dropped it when reducing the reference list. I think it is a very good paper, but it wouldn’t change our paper that much except for a more complete reference list. Do you have a specific request on what aspects of the paper we should cover?

Line 38 what specific deep muscles are you referring to, needs to be clear
remove the hyphen after deep muscles
line 39 needs rewording it seems unclear do you mean showed delay in muscle activation with perturbations?

\# The specific muscles are not that important given the context, IMO. But we added them and rephrased the sentence

Line 44 has this precise control been shown to be required in rowing, needs to be contextualised

\# Borghuis et al. (2008 -https://doi.org/10.2165/00007256-200838110-00002) elaborate on this in general. There does not seem to be much evidence (rather anecdotal, I guess). While this paragraph is not rowing-specific, the following is. No changes.

line 69 Not clear what this different approach is

\# explained the differences. Line 69-71

Line 71 what were the findings of the meta analysis referred to?

\# changed to „showed beneficial effects“

line 72 Another study evaluated PTT in high-level athletes.... what was the result, Not clear and not referenced

\# described results and referenced

line 74. you suggest the PPT could improve neuromuscular deficits in rowers. Going back to my comment about the title, it may be better to make a clear link between changing any potential deficits and the ability to influence or improve motor control rather than trunk stability

\# I took this as a premise (this is the whole point from paragraph 2). Improvement in deficits IS an improvement in motor control abilities for itself, IMO. So we suggest PTT -> improvement in neuromuscular deficits/motor control -> improvements in disability/pain. This might be wrong or not. This pathway could (or should) be tested in a mediation model. However, as critized for example by Lederman (2007 - <https://www.cpdo.net/Lederman_The_myth_of_core_stability.pdf>) , the proposed mechanisms are seldomly tested in our field. But running more and more analyses on our small data set would dramatically increase chances finding false positive results, so we refrain from that. And as stated elsewhere, our measure of balance might not be appropriate for this purpose.

**Study design**
line 82 as stated before no randomisation is a flaw and as such the current model may have introduced bias in the selection

\# Yes. No doubt, RCT is the gold standard. No doubt, we can have long arguments, whether non-randomized or 1-arm studies are worthwhile. The role of comparators is quite hard in general in LBP treatment research (Miller et al. (2021) - <https://doi.org/10.1007/s40279-021-01526-6>). While the causal treatment effect is constrained by the possible selection effect, the data from this study might be used with causal inference techniques (stratification, matching, etc.) in the future to reproduce causal relationships.

**Participants**
Line 92 More detail is required on what the usual routines of the CG were to make this reproducible
\# we described the very details according to CERT in the paper or the appendix (<https://mfr.de-1.osf.io/render?url=https://osf.io/3sqmw/?direct%26mode=render%26action=download%26mode=render>). We updated the descriptions for the CG, though.
Data Analysis
A power analysis should have been done here to determine the number of participants

\# we report our sample size determination honestly, as described in Lakens et al. (2021 - https://psyarxiv.com/9d3yf/ ). The justification for itself may not be the best, but we refrain from calculating post-hoc power and report as a priori. The examples in the discussion are meant to inform future research designs, like explained. No changes.

**Intervention**
line 106 was it any or all of the session that were supervised. What are sports therapists? are these physiotherapists or strength and conditioning trainers?

\# changed to „All of the total 16 group sessions were supervised by a physiotherapist and a sports scientist.”

Line 171 what is the significance of the reference to cost benefit as you did not measure this

\# described in more detail in the appendix and the discussion. This evaluation is recommended in the literature, so we did by choosing a small MIC and while discussing the results

Statistics
This is quite a detailed section but I am not clear why p values are not presented following tests like t test and ancova as this would be the usual convention and easier for the reader to follow.

\# it is a convention, but IMO not a good one. Reasons are eluded in the references. TL;DR: NHST can be misleading, especially in small sample sizes. Not worthwhile, testing hypotheses all the time. In a previous draft, the explanations for that decisions were even more detailed. An explanation of this approach in the strength training context is given by Schoenfeld et al. (2020) - https://doi.org/10.3389/fphys.2019.01571).

Results
Were t test performed on the demographic data? You state in line 197 The groups differ considerably in age and slightly in training volume but no p values are reported to indicate significance

\# As explained above, we don’t want to show p-values for „underpowered“ hypothesis tests. We added CI’s according to the style of our paper. So people can infer significance if they like…

Discussion
Line 301 you state Further research should incorporate specific balance measures (e.g. while sitting) to
302 evaluate the efficacy of PTT or similar interventions on trunk stability given the conjunction to injury
303 and low back pain [9,59] and sport-specific performance. Why was this not considered from the start as these seems key to the whole study

\# Not quite sure how to interpret the question. Do you mean we should have incorporated such specific measurements? We simply fell short when conducting the study. Non-specific adaptions (COP standing) were unlikely to happen considering to the findings in the literature (though, the evidence is not quite clear). But the results support at least this statement…
line 308 you state PTT aims to address neuromuscular deficits rather than maximal trunk strength itself. So why measure strength and not changes in motor control as suggested with the title change

\# the COP measurement was our surrogate for neuromuscular improvements. Not a wise choice as elaborated before. In contradiction, Arampatzis et al. found changes after 1 year of PTT in flexion/extension which is not yet addressed in the discussion

line 312 remove the word guess, non scientific language and 313 discussable?? if this a word

\# I guess it is: <https://www.lexico.com/definition/discussable>. Replaced „guess“ with „hypothesize“ but I think it is too formal in this context (don’t want to frame it as a worthwhile research question…). Replaced “discussable” with “debatable”.

line 324 you state PTT might work differently than other interventions, how might this be different, please expand

\# referenced the introduction

Practical Application
line 333 can you clarify what a usual trunk stabilization training routine is. This is a construct not clear in the paper

\# deleted that sentence and replaced it with the last from that paragraph

line 334 you state We observed high acceptance of our approach in elite rowers and positive feedback. Though this method is commonly used in many fields, the athletes in our study declared that many exercises were new to them. Where did this information come from? Not in the results

\# in the post questionnaire, we asked athletes questions about the feasibility and acceptance according to our training. While having too many outcome measures per se, I decided against implementing them in the methods and results section and only dedicating these lines to that aspect of our study. Would you suggest implementing that information in the methods/results section?

Limitations.
Apart from all the key ones mentioned in the start what does this mean line 350 athletes partly instructed themselves for
trunk stabilizing exercise, this would seem a major confounder to the whole study!

\# the athletes were instructed by coaches throughout the study but not BEFORE the study began. Does the sentence need rewording?

Conclusion
You state this PTT is possibly effective. I dont think you can state this as no meaningful difference was really found between the groups.

\# as mentioned above, we don’t think this estimate is not meaningful and is in line with the results from the SR/MA from Thornton et al. (2021) and even 2.5 points higher on average

Also line 358 you state Pain... which might be caused by seasonal effects. What does this mean ??

\# this is elaborated in lines 279-280
